# Factorial validity of an abbreviated Neighborhood Environment Walkability Scale for seniors in the Nurses’ Health Study

**DOI:** 10.1186/s12966-014-0126-8

**Published:** 2014-10-10

**Authors:** Heather A Starnes, Meghan H McDonough, Kosuke Tamura, Peter James, Francine Laden, Philip J Troped

**Affiliations:** California Polytechnic State University, 1 Grand Avenue, San Luis Obispo, CA 93407 USA; Purdue University, 800 W. Stadium Avenue, West Lafayette, IN 47907 USA; Harvard School of Public Health, 677 Huntington Avenue, Boston, MA 02115 USA; Brigham and Women’s Hospital Channing Laboratory, 181 Longwood Dr, Boston, MA 02115 USA; University Massachusetts Boston, 100 Morrissey Boulevard, Boston, MA 02125 USA

**Keywords:** Environment, Measurement, Walking, Older adults

## Abstract

**Background:**

Using validated measures of individuals’ perceptions of their neighborhood built environment is important for accurately estimating effects on physical activity. However, no studies to date have examined the factorial validity of a measure of perceived neighborhood environment among older adults in the United States. The purpose of this measurement study was to test the factorial validity of a version of the Abbreviated Neighborhood Environment Walkability Scale (NEWS-A) modified for seniors in the Nurses’ Health Study (NHS).

**Findings:**

A random sample of 2,920 female nurses (mean age = 73 ± 7 years) in the NHS cohort from California, Massachusetts, and Pennsylvania completed a 36-item modified NEWS-A for seniors. Confirmatory factor analyses were conducted to test measurement models for both the modified NEWS-A for seniors and the original NEWS-A. Internal consistency within factors was examined using Cronbach’s alpha. The hypothesized 7-factor measurement model was a poor fit for the modified NEWS-A for seniors. Overall, the best-fitting measurement model was the original 6-factor solution to the NEWS-A. Factors were correlated and internally consistent.

**Conclusions:**

This study provided support for the construct validity of the original NEWS-A for assessing perceptions of neighborhood environments in older women in the United States.

A growing body of built environment research has focused on associations between older adults’ perceptions of their neighborhood environment and physical activity [1-4]. However, data from a recent review of reviews underscores a lack of consistent findings for any environmental correlate of physical activity among older adults [5]. The consistent use of measures across studies could lead to firmer conclusions about the effects of environmental variables on physical activity in older adult populations. The development, testing and use of valid measures of individuals’ perceptions of their neighborhood environments are necessary in order to support inferences about effects of the built environment on physical activity. A key first step in the process of examining the construct validity of measures of perceptions is to examine their factorial validity [6]. Factorial validity is a form of construct validity that indicates whether constructs (i.e., factors) are unambiguously operationalized by the measured indicators [7].

Various measures of the perceived neighborhood environment have been used across different studies. One commonly used measure of individuals’ perceptions of their neighborhood environment is the Neighborhood Environment Walkability Scale (NEWS) and its abbreviated form (NEWS-A) [2-4,8-10]. These scales were designed to assess multiple dimensions of the perceived suitability of neighborhoods for walking with several Likert items per sub-scale and are consistent with a conceptual framework developed by Pikora and colleagues [11]. Results from two studies conducted in the United States have supported the factorial validity of NEWS-A among adults [9,10]. However, no known studies to date have examined this issue in older U.S. adults. Other less commonly used measures include single dichotomous (yes/no) items and single Likert items [1].

There may be important aspects of the neighborhood environment with respect to walking for older adults, which the commonly used NEWS and NEWS-A and less commonly used single items may not measure. For example, pedestrian and personal safety features of the neighborhood environment (e.g., lighting, sidewalk and crosswalk design, loitering teenagers, loose dogs, or vehicles crossing sidewalks to exit driveways) may be particularly salient to older adults with conditions that have impacted their balance, visual acuity, and physical reaction time. Therefore, the purpose of this study was to develop and test the factorial validity of a modified NEWS-A for seniors in a sample of older women in the United States.

## Methods

### Participants

Participants were selected from the Nurses’ Health Study (NHS) cohort, which started in 1976 with 121,701 married nurses aged 30 to 55 years living in 11 of the most populous U.S. states [12]. NHS investigators originally selected nurses because they anticipated that participants with a nursing education would be able to answer health-related questions with accuracy and would be likely to participate in a long-term study for several decades. NHS participants complete an extensive questionnaire every two years. The total size of the NHS cohort in 2008 in all U.S. states was 79,285 women, including women from California (n = 8,561), Massachusetts (n = 6,685), and Pennsylvania (n = 13,255). In 2008, a supplemental questionnaire that included a modified NEWS-A was mailed to a sample of 3,900 participants (4.92% of the cohort) who were randomly selected from early responders to the 2008 NHS biennial questionnaire. Three U.S. states, California, Massachusetts, and Pennsylvania, were selected to gain regional diversity from the more populous states in the Northeast and Western regions of the United States. The survey response rate was 84% (n = 3,275). Respondents were excluded from the analysis if any of the following conditions were met: 1) unable to walk (n = 75); 2) lived at current residence less than nine months of the year (n = 237); 3) lived at a different address during the four weeks prior to completing the supplemental survey (n = 26); 4) lived in an institutional setting (n = 6); or 5) had missing data on living situation (n = 13). Application of these criteria resulted in a final analytic sample of 2,920 women.

### Measures

In the biennial NHS questionnaire, participants reported their birth date (used to calculate age at time of completion of supplemental questionnaire), race (i.e., White, Black, Asian, American Indian/Alaska Native, Native Hawaiian), ethnicity (i.e., Hispanic, non-Hispanic), highest level of education (i.e., registered nurse degree, bachelors, masters, doctoral), current living situation (i.e., with spouse, with family, alone, nursing home, etc.), and walking limitations (i.e., yes/no, limited walking a block or several blocks). In the 2008 supplemental questionnaire, participants reported length of residence at current address (i.e., yes/no, lived at current address during the past four weeks and yes/no, lived 9 months or more of the year at the current address).

### Modified NEWS-a for seniors

The modified NEWS-A for seniors that was tested in this study consisted of seven subscales with 36 items. The original six subscales (24 items) from the original NEWS-A were included (i.e., access to destinations, street connectivity, infrastructure for walking, aesthetics, traffic safety, and personal safety) [9]. Nine additional items from an unpublished modified NEWS for seniors designed by the Senior Neighborhood Quality of Life study investigators were included to assess perceptions of personal safety, infrastructure for walking, and a seventh subscale of pedestrian safety. Current study investigators designed three additional items based on expert opinion (i.e., experts in urban planning, public health, health geography, and physical activity promotion) to assess perceptions of street connectivity and infrastructure for walking and biking. The added scale and items were intended to fit with the conceptual framework developed by Pikora and colleagues [11]. See Appendix for the full list of survey items. Participants rated their responses to items on a 4-point Likert scale (i.e., strongly disagree to strongly agree).

### Statistical analysis

Frequency tables and logistic regression were used to examine predictors of missing data for all items in the modified NEWS-A. An *a priori* 7-factor solution to the modified NEWS-A for seniors was tested using confirmatory factor analysis (CFA) based on a variance-covariance matrix. CFA was also conducted for the six original subscales in the original NEWS-A instrument [9]. We initially tested the CFA models to determine whether they fit, i.e., a confirmatory approach. Where there was misfit, we removed non-significant paths/factor loadings smaller than |.30| and which made sense theoretically to remove to estimate the most plausible model based on the data.

Model fits were assessed by root mean square error of approximation (RMSEA <0.06) [13], root mean square residual (RMSR <0.08) [13], comparative fit index (CFI >0.90) [14], and non-normed fit index (NNFI >0.90) [14]. Factor loadings were considered acceptable if they were greater than or equal to |0.30| [7]. If the *a priori* model fit was unacceptable, models were re-specified based on a review of factor loadings, modification indices, and conceptual meaningfulness. All analyses were conducted in 2012 using SAS 9.3 for UNIX.

## Findings

### Sample characteristics

Participants’ ages ranged from 61.5 to 88.4 years with a mean of 73.0 ± 6.9 years. The participants were mostly White (97.3%) and non-Hispanic (99.2%). The majority (66.9%) had a registered nurse (RN) degree only, 27.6% had a bachelor’s, master’s or doctoral degree, and 5.5% were missing information on education. Participants in California were slightly older than participants in Massachusetts and Pennsylvania (75.6 years vs. 71.8 and 71.6 years). A slightly lower percentage of participants in California were Caucasian compared to that in Massachusetts and Pennsylvania (93.1% vs. 99.8% and 99.2%). Education levels were lowest in Pennsylvania (76.3% had RN degree only), followed by Massachusetts (68.3%), and California (56.5%). Participants were similar in demographics (i.e., age, race, ethnicity, and education) to the full cohort (data not shown).

### Factorial validity of modified NEWS-a for seniors

Overall, less than 3% of the data at the case level in the modified NEWS-A for seniors were missing. Results of logistic regression analyses examining missing data points in relation to age, race, ethnicity, and education suggested there were no significant associations between demographic characteristics and missing data (corrected α .01 for multiple tests). Therefore, confirmatory factor analyses were conducted using full information maximum likelihood estimation with data from the final analytic sample of 2,920 participants. Furthermore, confirmatory factor analyses were tested using both full information maximum likelihood and list-wise deletion for missing data. Results were substantively similar; therefore, only models using full information maximum likelihood are reported.

The hypothesized *a priori* 7-factor solution to the modified NEWS-A for seniors did not adequately fit the observed data (model A in Table 1). Factor loadings on several items were less than |0.30| and several items had large standardized residuals, particularly items in a hypothesized infrastructure for walking/biking and pedestrian safety factors (data not shown). Model fit improved by collapsing items from these two factors into an infrastructure for walking factor and by the systematic removal of items with non-significant factor loadings. The final model for the modified NEWS-A for seniors was a 6-factor, 26-item solution (model B in Table 1). Factor loadings for items in the final model are presented in Table 2. Internal consistency estimates were high for five of the six modified NEWS-A factors (α ≥ 0.75) and were lowest for the street connectivity factor (α = 0.57) (Table 3). Moderate correlations were observed between the modified NEWS-A for seniors factors ‘access to destinations’, ‘street connectivity’, and ‘infrastructure for walking’ (r = 0.44 – 0.56) and between ‘aesthetics’, ‘traffic safety’, and ‘personal safety’ (r = 0.25 – 0.30) (Table 4, below the diagonal).

**Table 1:** **Measurement model fit indices for confirmatory factor analyses of modified NEWS-A for seniors and original NEWS-A among 2,920 NHS participants (aged 61–88 years) in California, Massachusetts, and Pennsylvania, 2008**

**Measurement model**	***χ*** ^**2**^	***df***	**CFI**	**NNFI**	**RMSEA**	**RMSR**
*A priori* modified NEWS-A for seniors^a^	8384.53	573	0.75	0.73	0.07	0.10
Final modified NEWS-A for seniors^b^	2557.13	284	0.91	0.90	0.05	0.05
*A priori* original NEWS-A^c^	2891.72	237	0.87	0.85	0.06	0.06
Final original NEWS-A^d^	1313.07	137	0.94	0.92	0.05	0.05

*Abbreviations:* *CFI* comparative fit index, *NNFI* non-normed fit index, *RMSEA* root mean square error of approximation, *RMSR* root mean square residual.

^a^7-factor solution, 36 items (24 original NEWS-A items and 12 additional items).

^b^6-factor solution, 26 items.

^c^6-factor solution, 24 items.

^d^6-factor solution, 19 items.

**Table 2:** **Standardized factor loadings in the final models of the modified NEWS-A for seniors and original NEWS-A among NHS participants in California, Massachusetts, and Pennsylvania, 2008**

**Factor and items**	**Modified**	**Original**
	**NEWS-A for seniors**	**NEWS-A**
Access to destinations factor		
Stores within easy walking distance	.81	.81
Many places within easy walking distance	.81	.82
Easy to walk to a transit stop	.65	.65
Street connectivity factor		
Short distance between intersections	.53	.52
Many alternative routes	.61	.64
Straight streets, not curvy	.51	*N/A*
Infrastructure for walking factor		
Sidewalks on most streets	.88	.92
Cars divide sidewalk and traffic	.71	.71
Grass/dirt strip divides sidewalk and traffic	.64	.64
Streets are well lit at night	.55	.54
Crosswalks have beeps	.31	*N/A*
Pedestrian signals give time to cross	.51	*N/A*
Curb cuts or ramps	.76	*N/A*
Aesthetics factor		
Trees along the streets	.41	.41
Interesting things to look at	.79	.79
Attractive natural sights, views	.86	.86
Attractive buildings, homes	.68	.68
Traffic safety factor		
Traffic makes it difficult to walk	.81	.76
Traffic speed is usually slow	.60	.64
Most drivers exceed the speed limit	.57	.60
Safe to walk in or along street	.62	*N/A*
Personal safety factor		
High crime rate	.78	.82
Crime makes it unsafe to walk during day	.68	.66
Crime makes it unsafe to walk during night	.77	.77
Unsafe alleys between buildings	.55	*N/A*
Loitering teenagers make it unsafe to walk	.63	*N/A*
Other items not in a factor		
Few cul-de-sacs	*Not included*	*Not included*
Walkers and bikers easily seen	*Not included*	*Not included*
Parking is difficult in shopping areas	*Not included*	*Not included*
Hilly streets	*Not included*	*Not included*
Major barriers to walking	*Not included*	*Not included*
Bicycle lanes or trails	*Not included*	*N/A*
Islands in the middle of the road	*Not included*	*N/A*
Have to cross busy streets to get to shops	*Not included*	*N/A*
Cars crossing sidewalks	*Not included*	*N/A*
Stray or loose dogs	*Not included*	*N/A*

Not included: Item not included in the final measurement model.

N/A: Item found only in modified NEWS-A for seniors, not in original NEWS-A.

See Appendix for full item wording.

**Table 3:** **Comparison of number of items, factor means, and internal consistency for factors in the final models of modified NEWS-A for seniors and original NEWS-A among NHS participants in California, Massachusetts, and Pennsylvania, 2008**

**Factor**	**No. of items**	**Mean (SD)**	**α**
Street connectivity - modified	3	2.68 (0.82)	.57
Street connectivity	2	2.73 (0.91)	.50
Infrastructure for walking - modified	7	2.15 (0.83)	.81
Infrastructure for walking	4	2.36 (0.97)	.78
Traffic safety - modified	4	2.86 (0.75)	.75
Traffic safety	3	2.83 (0.77)	.71
Personal safety - modified	5	3.72 (0.45)	.81
Personal safety	3	3.64 (0.55)	.79
Aesthetics^a^	4	3.28 (0.63)	.77
Access to destinations^a^	3	2.06 (0.97)	.80

^a^Subscales are the same in the original NEWS-A and modified senior NEWS-A.

**Table 4:** **Pearson correlations for modified NEWS-A for seniors and original NEWS-A factors among NHS participants in California, Massachusetts, and Pennsylvania, 2008**

**Factors**	**L1**	**L2**	**L3**	**L4**	**L5**	**L6**
Access to destinations (L1)	**1.00**	0.40	0.49	0.13	0.13	−0.15
Street connectivity (L2)	0.44	**0.90**	0.46	0.11	0.23	−0.11
Infrastructure for walking (L3)	0.56	0.51	**0.94**	0.09	0.20	−0.19
Aesthetics (L4)	0.13	0.06	0.11	**1.00**	0.26	0.23
Traffic safety (L5)	0.12	0.23	0.16	0.29	**0.95**	0.28
Personal safety (L6)	−0.17	−0.16	−0.23	0.25	0.30	**0.95**

The diagonal (in bold font) contains correlations between modified for seniors and original NEWS-A factors. Correlations among the modified NEWS-A for seniors factors are below the diagonal. Correlations among the original NEWS-A factors are above the diagonal.

### Factorial validity of original NEWS-A

The *a priori* 6-factor, 24-item solution to the original NEWS-A was not a good fit (model C in Table 1). A final 6-factor, 19-item solution that demonstrated adequate fit was achieved by removing five items (model D in Table 1). Four of the five removed items were treated as single items (i.e., not part of a NEWS-A factor) in previous studies [9,10] and assessed cul-de-sacs, parking in shopping areas, hilly streets, and major barriers to walking. The other item that was not included in the final solution assessed the visibility of walkers and bikers from homes. Significant factor loadings for the items in the final solution of the original NEWS-A are presented in Table 2. Factors in the final models for the original and modified NEWS-A had similar levels of internal consistency (Table 3). The patterns of correlations between factors were similar for the two instruments (Table 4).

## Conclusions

The purpose of this measurement study was to test the factorial validity of a modified NEWS-A for seniors in a large sample of older women in the United States. This paper may be the first to report on this aspect of validity for a measure of perceived neighborhood environment specifically in older U.S. adults. As more attention is devoted to examining associations between the built environment and physical activity among older adults, it is important to evaluate and report on the psychometric properties of the perceived environment measures used with this population. The results of this study suggest that the original NEWS-A has factorial validity for use among older women in the U.S. and that our modified NEWS-A for seniors did not add a salient dimension to the measure. A finding of particular importance was that items hypothesized to load on a new pedestrian safety factor were complex and not distinct from items that loaded on the original infrastructure for walking factor. This underscores the challenge of examining single aspects of environmental perceptions. Pedestrian safety and infrastructure for walking appear to be closely related concepts.

Few minor modifications to the original NEWS-A model were required to achieve acceptable fit with the data. For example, the removed item, “walkers and bikers on the street in my neighborhood can be easily seen by people in their homes” was hypothesized to load on the infrastructure for walking factor. However, the factor loading was below the threshold of 0.30. In previous studies of adults, this item had acceptable loadings of 0.61 and 0.46 [9,10]. These minor modifications made to the current NEWS-A model need to be confirmed with future research in older adults.

The final 6-factor NEWS-A model that was supported in this study was consistent with those reported in previous validation studies of NEWS-A among adults with both high and low socioeconomic status in the U.S. [9,10]. The current evidence suggests that the original NEWS-A [9] can be used to assess perceptions of neighborhood environment walkability among older women with educational backgrounds greater or equivalent to a registered nurse degree. All of the participants in the current study were practicing nurses in 1976 and they may not represent diverse women in other careers or with other employment histories.

Additionally, the results of this study may not generalize to other regions of the United States. Previous studies [9,10] were conducted in cities in the Northwestern region and Eastern regions of the U.S. and the current study was conducted in states in the Western and Northeastern regions of the U.S. It is not clear whether the NEWS-A model will generalize to older women living in the Southern and Midwestern regions. Further research is needed to address the generalizability of the NEWS-A measure in other regions and settings.

Another limitation of this study is that the findings may not be generalizable to women from diverse racial and ethnic groups or to older men. The majority of women in this study were non-Hispanic White. It is unknown whether the women in this study perceive their neighborhood walking environments in a similar manner as women with more diverse racial/ethnic backgrounds, residing in other U.S. states or regions, and with different education and employment histories. It is assumed though that the construct of walkability would be perceived similarly, but this assumption of factorial invariance needs to be tested in future research. Two notable strengths of this study are the regional variability of the sample (i.e., older women from two distinct U.S. regions) and the finding that a parsimonious walkability scale, the original NEWS-A, has factorial validity for use among older women in three U.S. states. In conclusion, the six subscales of the original NEWS-A appear valid for measurement of how older women perceive their neighborhood walking environments.

## Appendix

Items in the final model of the Modified NEWS-A for Seniors.

1. Access to destinations factor
  - Stores are within easy walking distance of my home.
  - There are many places to go within easy walking distance of my home.
  - It is easy to walk to a transit stop (bus, train) from my home.
2. Street connectivity factor
  - The distance between intersections in my neighborhood is usually short (100 yards or less; the length of a football field or less).
  - There are many alternative routes for getting from place to place in my neighborhood. (I don’t have to go the same way every time).
  - The streets in my neighborhood are straight. The do not have many curves.
3. Infrastructure for walking factor
  - There are sidewalks on most of the streets in my neighborhood.
  - Sidewalks are separated from road/traffic in my neighborhood by parked cars.
  - There is a grass/dirt strip that separates the streets from the sidewalks in my neighborhood.
  - My neighborhood streets are well lit at night.
  - The crosswalks in my neighborhood are designed for people who don’t see well because they have things like beeps that tell you when to cross.
  - Pedestrian signals in my neighborhood give me enough time to cross the road.
  - There are curb cuts (ramps) that go from sidewalk level to road level in my neighborhood (for example, at the end of sidewalks).
4. Aesthetics factor
  - There are trees along the streets in my neighborhood.
  - There are many interesting things to look at while walking in my neighborhood.
  - There are many attractive natural sights in my neighborhood (such as landscaping, views).
  - There are attractive buildings/homes in my neighborhood.
5. Traffic safety factor
  - There is so much traffic along streets in my neighborhood that it makes it difficult or unpleasant to walk.
  - The speed of traffic on most streets in my neighborhood is usually slow (30 mph or less).
  - Most drivers exceed the posted speed limits while driving in my neighborhood.
  - It is safe to walk in the street or along the side of the street in my neighborhood.
6. Personal safety factor
  - There is a high crime rate in my neighborhood.
  - The crime rate in my neighborhood makes it unsafe to go on walks during the day.
  - The crime rate in my neighborhood makes it unsafe to go on walks at night.
  - There are alleys between buildings that make it unsafe to walk in my neighborhood.
  - There are teenagers hanging out that make it unsafe to walk in my neighborhood.
7. Other items not in factor
  - The streets in my neighborhood do not have many cul-de-sacs (dead-end streets).
  - Walkers and bikers on the streets in my neighborhood can be easily seen by people in their homes.
  - Parking is difficult in local shopping areas.
  - The streets in my neighborhood are hilly, making my neighborhood difficult to walk in.

## References

[1] Michael Y, Beard T, Choi D, Farquhar S, Carlson N (2006). Measuring the influence of built neighborhood environments on walking in older adults. J Aging Phys Act.

[2] King D (2008). Neighborhood and individual factors in activity in older adults: results from the neighborhood and senior health study. J Aging Phys Act.

[3] de Melo LL, Menec V, Porter MM, Ready AE (2010). Personal factors, perceived environment, and objectively measured walking in old age. J Aging Phys Act.

[4] Shigematsu R, Sallis JF, Conway TL, Saelens BE, Frank LD, Cain KL, Chapman JE, King AC (2009). Age differences in the relation of perceived neighborhood environment to walking. Med Sci Sports Exerc.

[5] Bauman A, Reis RS, Sallis JF, Wells JC, Loos RJ, Martin BW, Lancet Physical Activity Series Working Group (2012). Correlates of physical activity: why are some people physically active and others not?. Lancet.

[6] Dimitrov DM (2010). Testing for factorial invariance in the context of construct validation. Meas Eval Couns Dev.

[7] Tabachnick BG, Fidell LS (2007). Using Multivariate Statistics.

[8] Saelens BE, Sallis JF, Black JB, Chen D (2003). Neighborhood-based differences in physical activity: an environment scale evaluation. Am J Public Health.

[9] Cerin E, Saelens BE, Sallis JF, Frank LD (2006). Neighborhood Environment Walkability Scale: validity and development of a short form. Med Sci Sports Exerc.

[10] Cerin E, Conway TL, Saelens BE, Frank LD, Sallis JF (2009). Cross-validation of the factorial structure of the Neighborhood Environment Walkability Scale (NEWS) and its abbreviated form (NEWS-A). Int J Behav Nutr Phys Act.

[11] Pikora T, Giles-Corti B, Bull F, Jamrozik K, Donovan R (2003). Developing a framework for assessment of the environmental determinants of walking and cycling. Soc Sci Med.

[12] Belanger CF, Hennekens CH, Rosner B, Speizer FE (1978). The Nurses’ Health Study. Am J Nurs.

[13] Hu L, Bentler P (1999). Cutoff criteria for fit indexes in covariance structure analysis: conventional criteria versus new alternatives. Struct Equ Modeling.

[14] Bentler PM (1990). Comparative fit indexes in structural models. Psychol Bull.

